# Conservation in a cup of water: estimating biodiversity and population abundance from environmental DNA

**DOI:** 10.1111/j.1365-294X.2012.05600.x

**Published:** 2012-06

**Authors:** David M Lodge, Cameron R Turner, Christopher L Jerde, Matthew A Barnes, Lindsay Chadderton, Scott P Egan, Jeffrey L Feder, Andrew R Mahon, Michael E Pfrender

**Affiliations:** * Environmental Change Initiative, University of Notre DameNotre Dame, IN 46556, USA; † Department of Biological Sciences, University of Notre DameNotre Dame, IN 46556, USA; ‡ Great Lakes Project, The Nature ConservancyNotre Dame, IN, USA; § Advanced Diagnostics and Therapeutics Initiative, University of Notre DameNotre Dame, IN 46556, USA; ¶ Genomics Core Facility, University of Notre DameNotre Dame, IN 46556, USA

**Keywords:** biodiversity assessment, biosecurity, eDNA, invasive species, natural resource management, pyrosequencing, qPCR

## Abstract

Three mantras often guide species and ecosystem management: (i) for preventing invasions by harmful species, ‘early detection and rapid response’; (ii) for conserving imperilled native species, ‘protection of biodiversity hotspots’; and (iii) for assessing biosecurity risk, ‘an ounce of prevention equals a pound of cure.’ However, these and other management goals are elusive when traditional sampling tools (e.g. netting, traps, electrofishing, visual surveys) have poor detection limits, are too slow or are not feasible. One visionary solution is to use an organism’s DNA in the environment (eDNA), rather than the organism itself, as the target of detection. In this issue *of Molecular Ecology*, Thomsen *et al.* (2012) provide new evidence demonstrating the feasibility of this approach, showing that eDNA is an accurate indicator of the presence of an impressively diverse set of six aquatic or amphibious taxa including invertebrates, amphibians, a fish and a mammal in a wide range of freshwater habitats. They are also the first to demonstrate that the abundance of eDNA, as measured by qPCR, correlates positively with population abundance estimated with traditional tools. Finally, Thomsen *et al.* (2012) demonstrate that next-generation sequencing of eDNA can quantify species richness. Overall, Thomsen *et al.* (2012) provide a revolutionary roadmap for using eDNA for detection of species, estimates of relative abundance and quantification of biodiversity.

## Rapid development and application of eDNA approaches

**Fig. 1:**
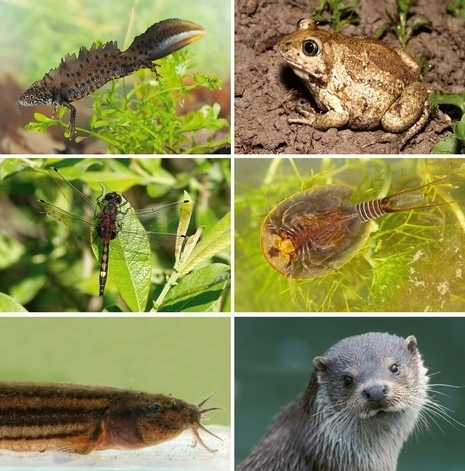
The six species targeted in Thomsen *et al.* (2012). From left to right and top to bottom: Great crested newt (*Triturus cristatus*), adult Common spadefoot toad (*Pelobates fuscus*), adult Large white-faced darter (*Leucorrhinia pectoralis*), Tadpole shrimp (*Lepidurus apus*), European weather loach (*Misgurnus fossilis*) and Eurasian otter (*Lutra lutra*). (Copyright: top left and middle right, ©http://www.deschandol-sabine.com; bottom right, © Gerhard Schulz/Polfoto; all other, © Lars L. Iversen).

**Fig. 2:**
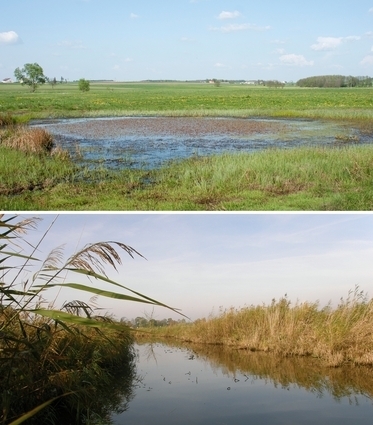
Examples of sampling sites in Thomsen *et al.* (2012). Top: Pond habitat for the amphibian species. Bottom: Running water habitat for the European weather loach. (Copyright: top, © Lars L. Iversen; bottom, © Philip Francis Thomsen).

Recent applications of eDNA have surprised some environmental managers because they seemed to emerge abruptly from the research phase (Darling & Mahon 2011). Yet the research that produced these tools illustrates typical and incremental scientific progress. The term ‘environmental DNA’ originates from microbiology (Ogram *et al.* 1987) and generally means DNA extracted from an environmental sample without isolating the target organism; for macrobiota, an entire organism is often not even present in the sample. Research targeting ‘macrobial’ eDNA began with detection of plant DNA in soil (Paget *et al.* 1998), with the first metagenetic approach (*sensu*Creer *et al.* 2010) to eDNA also applied to soil (Willerslev *et al.* 2003). The first application of macrobial eDNA analysis in an aquatic environment detected human, cow, pig and sheep DNA in river water (Martellini *et al.* 2005), with the first aquatic metagenetic approach aimed at riverine fishes (Minamoto *et al.* 2011). In this issue, Thomsen *et al.* (2012) apply the most current techniques, quantitative real-time PCR (qPCR) and next-generation sequencing, to demonstrate compellingly the power of the eDNA approach (Figs 1 and 2).

Specifically, results from qPCR provide an index of population size, which is a very important advance over PCR (Thomsen *et al.* 2012). In addition, qPCR has a lower detection threshold than traditional sampling tools, probably even lower than PCR because of the generally greater sensitivity of qPCR (Thomsen *et al.* 2012). Finally, using qPCR, Thomsen *et al.* (2012), expanding on other recent studies (Dejean *et al.* 2011), observed that the rapid degradation of eDNA in surface water means that the detection of eDNA indicates the very recent presence of aquatic species. In 80L tank experiments with a toad and a newt species, the longest that eDNA remained detectable at the highest organism density after removal of all amphibians was between 9 and 15 days (Thomsen *et al.* 2012).

The application by Thomsen *et al.* (2012) of next-generation sequencing shows how to move forward from targeted surveillance of one, or a handful of species, to more accurate estimates of species richness.

## New eDNA tools in the toolbox to facilitate management goals for species and ecosystems

Some prominent early applications of environmental DNA have involved the detection of faecal pollution (Martellini *et al.* 2005) and invasive species (Ficetola *et al.* 2008; Jerde *et al.* 2011). With invasive species, finding incipient populations early provides managers with options to act before a harmful species achieves high abundance (Robinson *et al.* 2011). Similarly, identifying and protecting habitats important to the persistence of biodiversity is daunting, particularly if threatened or endangered species are difficult to detect (Goldberg *et al.* 2011) or restrictions prevent sampling efforts that risk harm to individual organisms (Beja-Pereira *et al.* 2009). Thomsen *et al.* (2012) and other recent papers (Pfrender *et al.* 2010) point the way towards the power of eDNA for identifying habitats critical to protected species, and for assessing biodiversity for conservation, remediation and restoration efforts.

## Trajectory of aquatic eDNA research: extracting more information more rapidly

We believe that Thomsen *et al.* (2012) represents a macrobial eDNA research agenda that will proceed rapidly along at least two trajectories: species-specific population surveillance and monitoring; and metagenetic detection of multiple species simultaneously. Massively parallel technologies like next-generation sequencing and microarrays can measure biodiversity across broad taxonomic scales. In comparison with microbial metagenetics, macrobial metagenetics benefits from a much smaller number of taxa, more reliable species boundaries and a considerable public database linking taxonomic and genetic identities. Macrobial metagenetics is already transforming the science of biodiversity assessment (Anderson-Carpenter *et al.* 2011).

However, one of the striking gaps in this rapidly growing field is the dearth of knowledge about how field and laboratory protocols influence the detection of eDNA (Goldberg *et al.* 2011), and how different environmental conditions affect the production, degradation and detection of eDNA. For example, a wide range of protocols have been reported for field sampling (e.g. number and volume of water samples), filtration (e.g. precipitation vs. various filters), DNA extraction (e.g. different kits and protocols), primer design and testing, PCR (e.g. number of reactions) and confirmation of species specificity (e.g. cloning, sequencing). The latter could be an underappreciated problem when it is critical to resolve among closely genetically related taxa that could have vastly different repercussions for management and/or biocontrol (Funk & Omland 2003). Experiments to systematically compare protocols are urgently needed. For laboratory protocols, adherence to the minimum information reporting guidelines for qPCR and metagenetics (Taylor *et al.* 2008) would at least make it more possible to compare protocols among publications even if the specific effects of different protocols were unknown.

Environmental DNA analysis is already an essential and influential tool in water quality monitoring, the early detection of invasive and other harmful species and the surveillance of imperilled species. With further refinements and comparisons of field and laboratory protocols, eDNA analysis will provide more information on taxonomic diversity and population abundance and find wider applications in environmental science research. Thomsen *et al.* (2012) give us confidence that: (i) eDNA analysis is applicable across broad taxonomic boundaries; (ii) the presence of eDNA indicates the recent presence of organisms; (iii) we can expect to learn more and more about population abundance with qPCR-based eDNA analysis; and (iv) next-generation sequencing of eDNA will yield increasingly accurate estimates of species richness. With eDNA, a lot can be learned from a cup of water.
